# Animal Detection in Natural Images: Effects of Color and Image Database

**DOI:** 10.1371/journal.pone.0075816

**Published:** 2013-10-10

**Authors:** Weina Zhu, Jan Drewes, Karl R. Gegenfurtner

**Affiliations:** 1 School of Information Science, Yunnan University, Kunming, China; 2 Department of Psychology, Giessen University, Giessen, Germany; 3 Kunming Institute of Zoology, Chinese Academy of Science, Kunming, China; 4 Centre for Vision Research, York University, Toronto, Canada; University of Leuven, Belgium

## Abstract

The visual system has a remarkable ability to extract categorical information from complex natural scenes. In order to elucidate the role of low-level image features for the recognition of objects in natural scenes, we recorded saccadic eye movements and event-related potentials (ERPs) in two experiments, in which human subjects had to detect animals in previously unseen natural images. We used a new natural image database (ANID) that is free of some of the potential artifacts that have plagued the widely used COREL images. Color and grayscale images picked from the ANID and COREL databases were used. In all experiments, color images induced a greater N1 EEG component at earlier time points than grayscale images. We suggest that this influence of color in animal detection may be masked by later processes when measuring reation times. The ERP results of go/nogo and forced choice tasks were similar to those reported earlier. The non-animal stimuli induced bigger N1 than animal stimuli both in the COREL and ANID databases. This result indicates ultra-fast processing of animal images is possible irrespective of the particular database. With the ANID images, the difference between color and grayscale images is more pronounced than with the COREL images. The earlier use of the COREL images might have led to an underestimation of the contribution of color. Therefore, we conclude that the ANID image database is better suited for the investigation of the processing of natural scenes than other databases commonly used.

## Introduction

The visual system has a remarkable ability to extract categorical information from complex natural scenes, and the recognition of objects and scenes appears to be instantaneous and effortless. When we view an image, we can usually understand what is displayed very quickly and easily, and we do it countless times every day. It was shown that the complex processing needed to perform an object categorization task can be extremely fast in humans [1]. The speed of processing is particularly remarkable when considering that responses in early visual areas are dominated by rather elementary image features. For example, neurons in primary visual cortex (V1) respond best to oriented edges, movement or color. Only a few synapses further, in inferotemporal cortex, neurons are already selective to objects and give differential responses to different faces or persons [2], [3], [4], [5], [6], [7].

**Figure 1:**
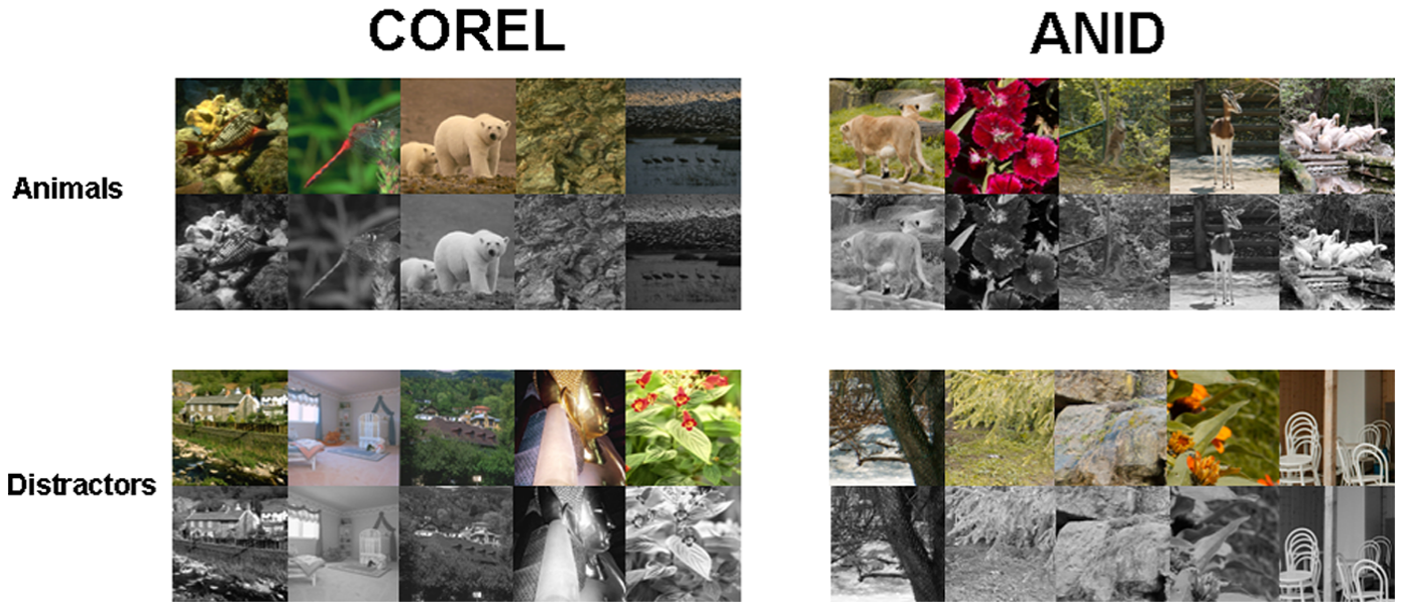
Sample images: animal (top) and non-animal (bottom) images of the Corel (left) and ANID databases (right); both color and gray-scale versions are shown.

Evidence for “ultra-fast” processing in the visual system mainly comes from EEG-recordings in manual response tasks and response times of saccadic eye movements [1], [8]. Simon Thorpe and colleagues reported that their observers were capable of detecting animals within novel natural scenes with remarkable speed and accuracy. In a manual go/no-go animal categorization task images were only briefly presented (20 ms) and already 150 ms after stimulus onset the no-go trials showed a distinct frontal negativity in the event related potentials (ERPs). Median reaction times (RTs) showed a speed-accuracy trade-off but for RTs as short as 390 ms observers were already approx. 92% correct (increasing to 97% correct for 570 ms) [1]. In a more recent study Kirchner & Thorpe [8] found that saccadic latencies in a 2-AFC (two alternatives-forced-choice) task were even shorter than the manual reaction times. Subjects on average took 228 ms to indicate which one of two images contained an animal. The shortest reaction times where subjects could reliably identify the animal image were on the order of 120 ms – saccadic latencies for more elementary discriminations (e.g. orientation of a line) are only slightly faster [9], [10], [11], [12], or not faster at all [13]. This leaves very little time for visual cortical processing, other than a feed-forward sweep through the ventral stream.

**Figure 2:**
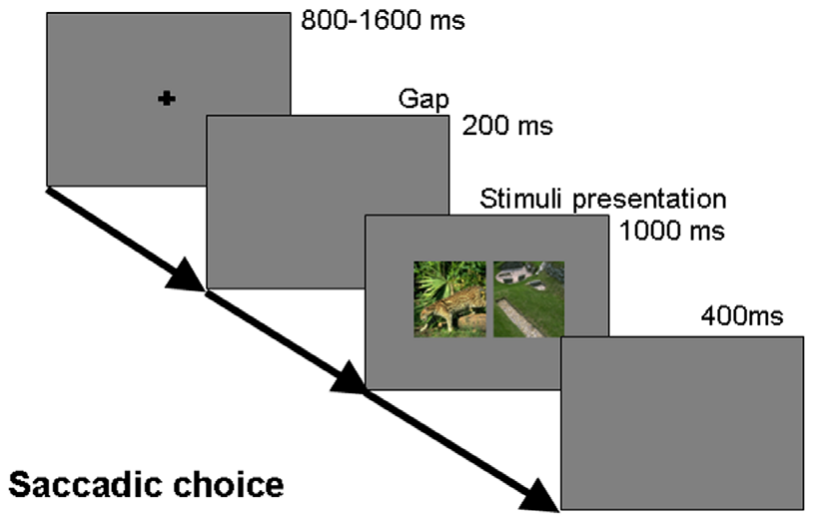
Schematics of the paradigm used in experiment 1. Participants were instructed to direct their gaze towards the target image as quickly as possible.

This ultra-rapid and accurate object detection has been found under various complex stimulus conditions, besides the detection of objects embedded in natural scenes, such as animals, food, fish and trees [14], [15], there are artificial objects such as various vehicles containing cars, aircraft, boats, etc. [16]. However the role of low level image features for the recognition of objects in natural scenes is still very controversial [17], [18], [19], [20], [21]. On the one hand, in Thorpe, Fize, and Marlot 's research, they suggested that the presence of ‘no-go’ specific frontal activity at 150 ms implies that the visual processing for task performance has been completed before this time, and much of this processing is based on essentially feed-forward mechanisms [1]. Other studies also found large effects with onsets at about 150 ms in the animal detection task [16], [22]. Furthermore, Kirchner and Thorpe suggested low level differences between target and distractor images were unable to account for these exceptionally fast responses [8]. On the other hand, Johnson and Olshausen suggested that the early component (around 135 ms) of neural activity is not correlated to recognition but low-level feature difference between images, and the neural signatures of recognition have a substantially later and variable time of onset (150–300 ms) [23].

**Figure 3:**
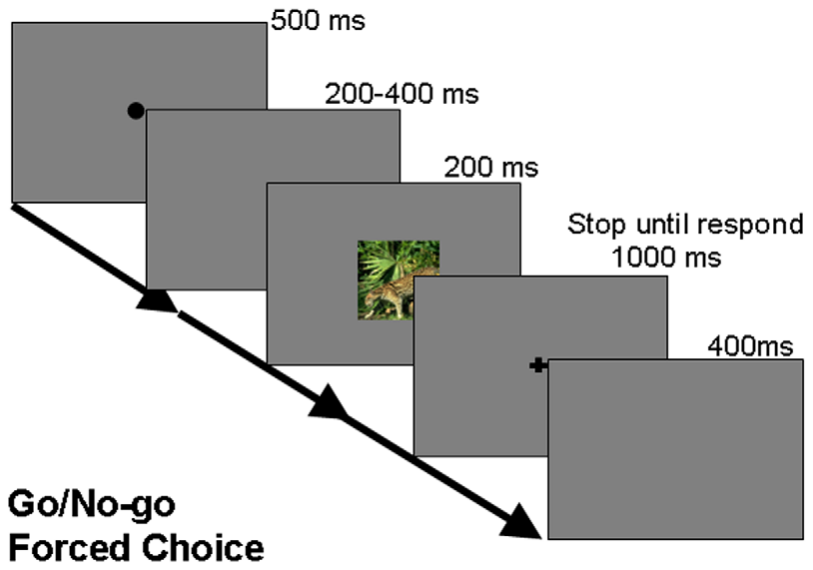
Schematic of the paradigm used in experiment 2. In the go/nogo task, participants were instructed to release a button when a target image was shown, and to hold on to the button otherwise. In the forced choice task, participants were asked to press different buttons for animal and non-animal images.

Most of the research about the animal detection in natural scene used professional photographs from the Corel Stock Photo Library (COREL) which contains images taken by professional photographers. Thus, the animal images contain animals in sharp focus at the center of the image, in front of a mostly blurry background. The distractor images mostly contain landscapes, including man-made objects, which are all in sharp focus [20]. This selection of the stimulus material might have affected stimulus processing. Aude Oliva, Antonio Torralba and colleagues suggested that observers might guide their decision using a form of contextual information. Instead of recognizing the animal in a scene, observers use other cues (called “proxies”) that are correlated with the presence of an animal. In this case, it was mainly the spatial frequency content of the scenes under study [24]. In order to remedy that situation, an image database called All Natural Images Database (ANID) (http://www.allpsych.uni-giessen.de/ANID/) was newly developed to overcome the drawbacks of the COREL database. The pictures were taken with high resolution with the animal occupying a small standard region at the center of the image. This way, the images can be shifted around so that the animal can take up different positions in the image, or in the extreme case be shifted out of the image altogether. The advantage is that all contextual cues can be excluded, because the animals are just as likely to be present given the background of the distractor images. The kind of “photographer-induced” proxy strategy suggested fails in these images, yet observers were still capable of ultrafast animal recognition [20], [25].

**Figure 4:**
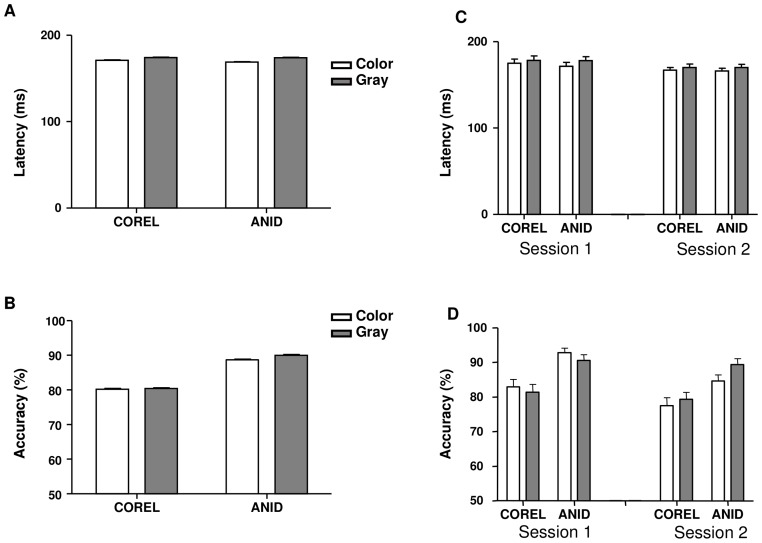
Performance of experiment 1. Saccade latency (A) and Accuracy (B) of image database (COREL vs. ANID) × image hue (color vs. gray-scale), in two sessions (C, D). Error bars represent 1 s.e.m.

Furthermore, context (or global scene) congruence has generally been shown to have a significant effect on classification accuracy and speed [26], [27]. In the ANID database, images of animals were taken in their natural habitats or in zoo environments coming close to the natural habitat of the animals. Thus the animals from ANID have a more contiguous background than in previous studies using the COREL database. These stimuli maintain animal/background integrity and thereby avoid any kind of “cut out” or “cut and paste” effect [25]. While a host of literature exists regarding image databases in computer vision (see e. g. [28], [29]), there is little direct research about the role of image databases in visual perception, including rapid animal detection performance. Thus we used this new natural image database as a comparison to the COREL images.

**Figure 5:**
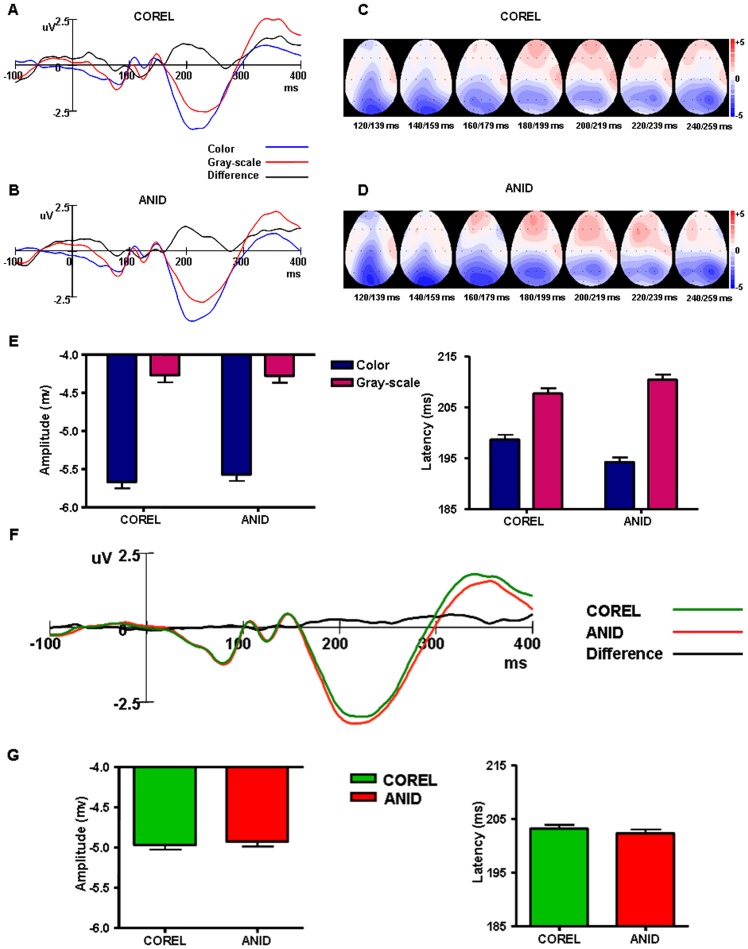
Grand average ERP waveforms of experiment 1. Frontal areas: F3, F4, F7, F8, FZ, FP1, FP2. ERP waveforms elicited by Color and Gray-scale images and their difference waveforms in COREL (A) and ANID (B) databases separately. Topographic maps for the difference waves in COREL (C) and (D). The ERPs were integrated across 20 ms time windows from 120 ms to 259 ms. Maps are viewed from above, with the nose pointing upwards. (F) ERP waveforms of COREL and ANID and their difference waveforms at frontal areas. (E) and (G) Statistics of N1 amplitudes (left) and latency (right). Error bars represent 1 s.e.m.

We also investigated the role of another low level feature, color, for object recognition. The role of color information for recognizing scenes and objects has been somewhat elusive so far. The results have been quite different depending on the particular task. While many researchers have found great advantages of color in scene and object recognition, no such effect was obtained for the reaction times in the animal detection experiments [14].

**Figure 6:**
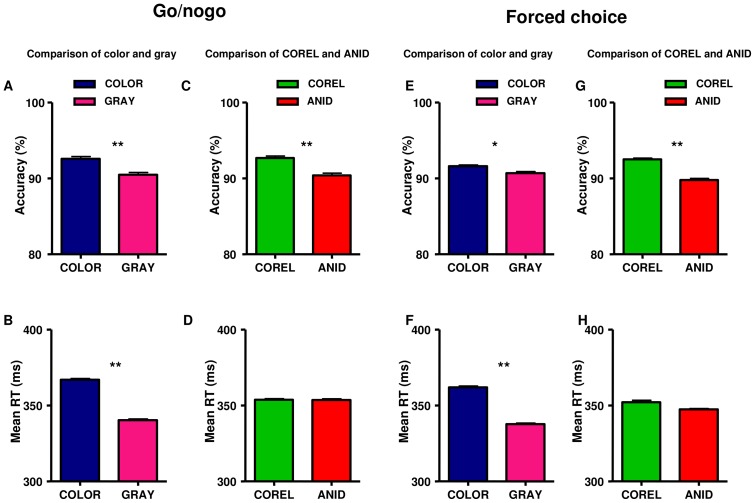
Behavioral performance of experiment 2. Accuracy (top) and Response Time (bottom) were compared between color and gray-scale stimuli (left) and between COREL and ANID databases (right) in go/nogo and forced choice task. Error bars represent 1 s.e.m.

Color provides humans with extraordinarily rich sensory access to the surrounding environment, for example to detect ripe fruit and distinguish young leaves against a background of mature leaves [30]. And it is well established that color information plays an important role in early visual processing [31], [32]. Color has been shown to aid image segmentation [33] and object recognition [34]. Image segmentation is typically used to locate objects and boundaries (lines, curves, etc.) in images, which help to determine “what” is “where”. Moller and Hurlbert demonstrated an early contribution of region-based processes to segmentation by color [33]. Gegenfurtner and Rieger used a delayed match-to-sample task to test the role of color vision in the recognition of briefly presented images of natural scenes. Recognition accuracy was higher for color images of natural scenes than for luminance-matched black and white images, and color information contributed to both very early and later stages of the recognition process [34]. This result concluded that color vision, whose processing starts at the very earliest stages of analysis, helps us to recognize things faster and to remember them better. More studies suggested that color facilitate recognition memory on a sensory level, at encoding, by improving edge detection and segmentation as well as on a cognitive level by being bound as a part of the memory representation [34], [35]. In general, these studies indicate that color helps to see things quicker, and to remember them better.

However, some studies found little effects of color for target detection in natural scenes [36], [37]. Fei-Fei et al. using a dual-task paradigm, whose peripheral task is a go/no-go detection task with a speeded response to detect animal targets, found that subjects performed equally well with grayscale and colored stimuli in briefly presented scenes [36]. Delorme et al. had both humans and rhesus monkeys perform a go/no-go detection task requiring a speeded response to detect targets on briefly flashed images (32 ms). They found that both accuracy and response times were virtually the same for grayscale and colored targets [37]. In contrast, when the response time was slower (>400 ms) than the ultra-rapid task (characterized by response times less than 360 ms in humans), color seems to have a more decisive role in scene categorization tasks [38], [39], [40]. The subjects were typically faster and more accurate when color diagnostic scenes were presented in original coloring rather than in grayscale [39], [41]. The conclusion from these studies seems to be that color information is available at a very early level, but that this information is not available to guide quick reactions.

The aim of our study is to investigate two aspects of low-level visual processing (color and database) on object recognition and its neural basis. In addition to behavioral data (reaction time and accuracy), we recorded the event-related potentials (ERPs) to obtain a more direct measure of the neural processes related to rapid scene categorization. Based on the excellent temporal resolution of neural events, ERPs have been regarded as neural manifestations of specific cognitive functions, reflecting brain activity from synchronously active populations of neurons [42]. ERPs can reveal, at much finer temporal resolution (millisecond range), the time course of neural processes underlying categorization [1], [23]. For example, Thorpe and his colleagues studied the processing time for natural scenes and found that the difference between go and no-go trials develops roughly 150 ms by ERP [1]. Importantly, the timing of this ERP component onset is constant across trials [23] and therefore is not correlated with the subsequent reaction time. The latency of this component is also constant for both novel and previously learned images [43]. Thus it is would be interesting to study the role of color and database in the scene categorization by ERP. No previous ERP studies have attempted to measure the neural basis for the color and database role in rapid categorization of natural scenes, thus, we used ERP to investigate: how and when these low level image features affect the processing of ultra-rapid animal detection in natural scene.

**Figure 7:**
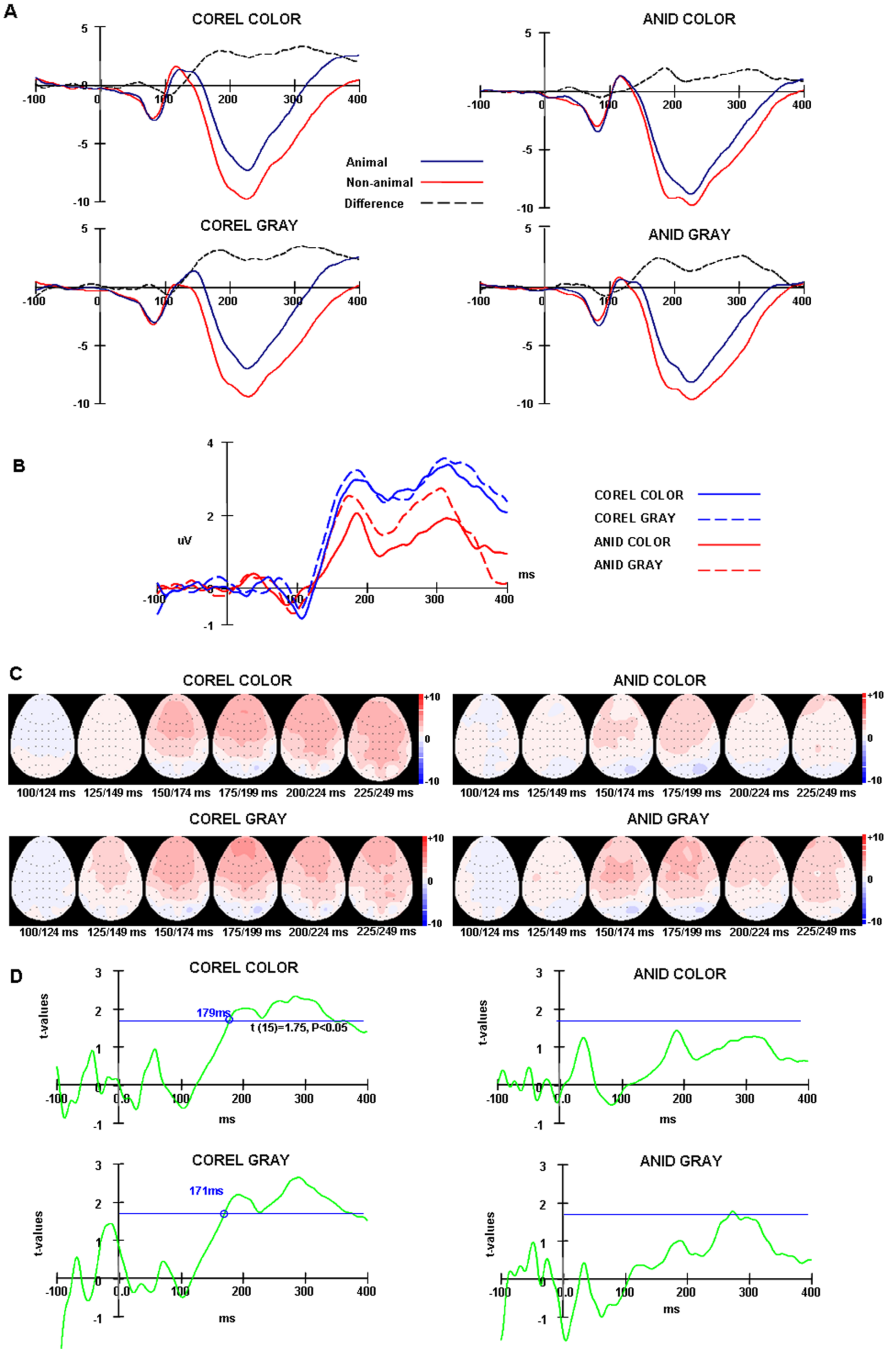
Grand average ERPs of the go/nogo task in experiment 2. (A) Animal and non-animal stimuli ERPs and their difference waveforms of COREL and ANID for color and gray-scale images from frontal areas (FP1, FP2, F3, F4, F7, F8, FZ). (B) The difference waveforms between animal and non-animal stimuli in four conditions. (C) Topographic maps for the difference waves in four conditions. The ERPs were integrated across 20 ms time windows from 120 ms to 259 ms. Maps are viewed from above, with the nose pointing upwards. (D) Paired t-test at each time point of the difference ERPs between −100 ms to 400 ms at frontal areas (n = 16, *t* (15)  = 1.75, *p*<0.05).

In Kirchner and Thorpe's study, eye movements instead of button presses were used: human observers have been shown to be capable of deciding which of two simultaneously presented natural scenes contains a target object in even less than 150 ms. On average, when using saccadic choice paradigms, human observers have been found to perform rapid animal detection with mean latencies around 200 ms, while maintaining accuracy ratings of 80% and up to 95% (Kirchner & Thorpe, 2006). Thus, in our study, different response paradigms and modalities were used (go/nogo vs. forced-choice; saccade vs. button presses) to test the effects of color and database in animal detection.

**Figure 8:**
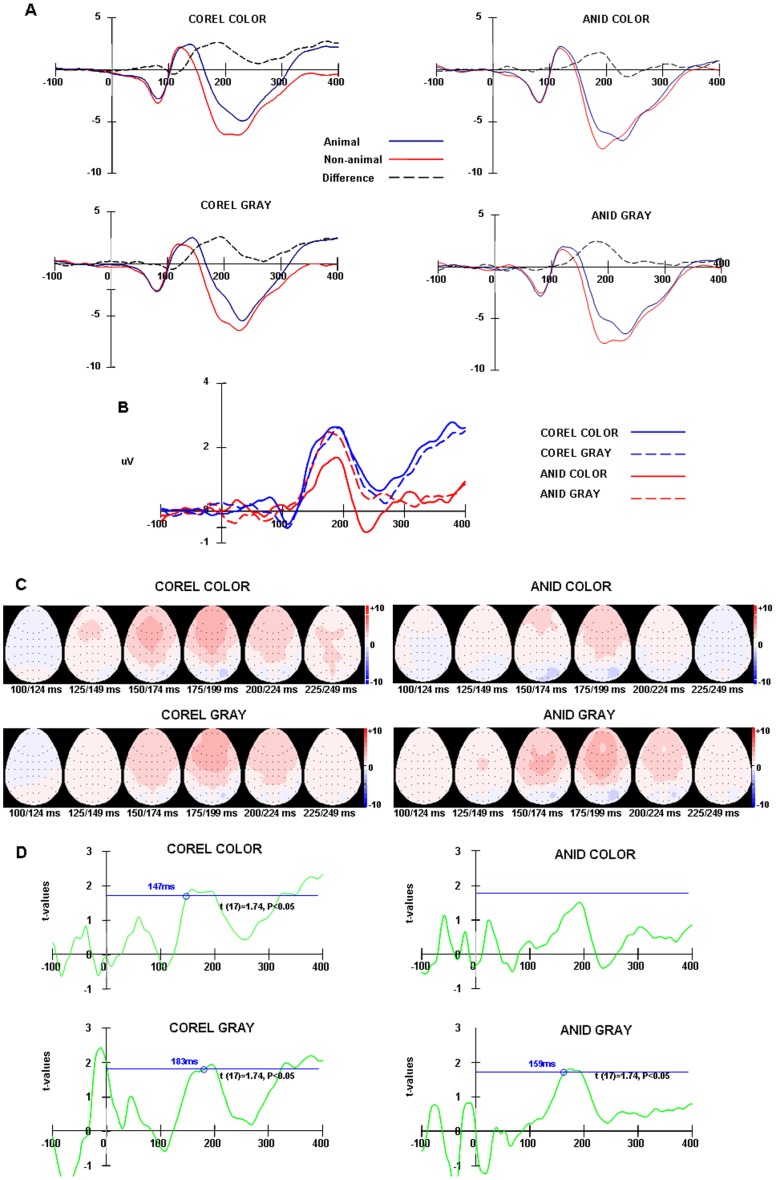
Grand average ERPs of the forced choice task in experiment 2. (A) Animal and non-animal stimuli ERPs and their difference waveforms of COREL and ANID for color and gray-scale images from frontal areas (FP1, FP2, F3, F4, F7, F8, FZ). (B) The difference waveforms between animal and non-animal stimuli in four conditions. (C) Topographic maps for the difference waves in four conditions: The ERPs were integrated across 20 ms time windows from 120 ms to 259 ms. Maps are viewed from above, with the nose pointing upwards. (D) Paired t-test at each time point of the difference ERPs between −100 ms to 400 ms at frontal areas (n = 18, *t* (17)  = 1.74, *p*<0.05).

## Methods

### Ethics statement

All experiments were approved by the local Ethics Committee of the Giessen University or Kunming Institute of Zoology, Chinese Academy of Sciences, and performed according to the principles expressed in the Declaration of Helsinki. All participants were informed about the procedure of the experiment. Written informed consent was obtained from all participants.

**Figure 9:**
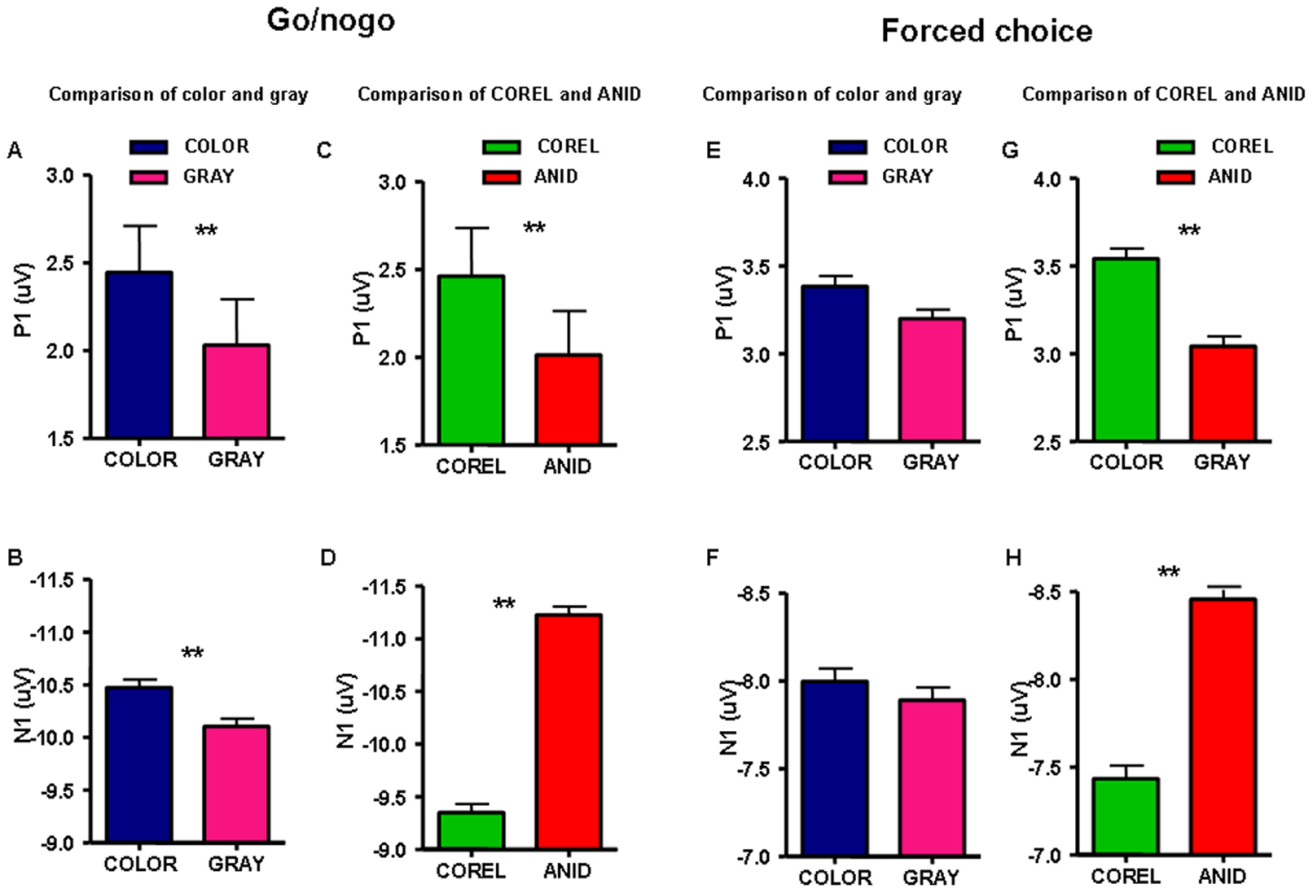
Comparison of ERP characteristics of experiment 2. P1 (top) and N1 (bottom) amplitude at frontal areas (FP1, FP2, F3, F4, F7, F8, FZ) were compared between color and gray-scale stimuli (left) and between COREL and ANID databases (right) in go/nogo and forced choice task. Error bars represent 1 s.e.m.

### Subjects

34 subjects participated in experiment 1 (10 male, 24 female, aged 18–34: mean  = 23, SD  = 3.01). 18 subjects were tested on color images, and the remaining 16 subjects on gray-scale images. The participants were students recruited from Giessen University and were paid hourly for their participation.

Another 34 subjects participated in experiment 2 (17 male, 17 female, aged 20–26: mean  = 22.3, SD  = 1.39), who did not take part in the experiment 1. 16 subjects performed in a go/nogo task, and another 18 subjects performed in a forced choice task. The participants were students recruited from Yunnan University and were paid hourly for their participation.

All participants had normal or corrected-to-normal vision and were naive to the purpose of the experiment.

### Stimuli

1200 images were used in the experiments. Six hundred images were selected from the Corel Stock Photo Libraries (COREL) [44], and another 600 images were selected from the All Natural Images Database (ANID) (http://www.allpsych.uni-giessen.de/ANID).

Many important studies involving rapid animal detection used images drawn from the COREL database [23], [43], [45], [46]. The center objects (animals) in the COREL database frequently cover a large portion of the available image area, leaving relatively little target-free surround [25]. The animal and non-animal stimuli of the COREL database were therefore collected from independent images, which may have resulted in systematic differences between animal and non-animal stimuli [20]. In contrast, the newly collected ANID database contains larger images with large, contiguous, yet animal-free surround. This enabled us to crop the animal and non-animal images from the same scenes in the ANID database. In order to compare with the COREL database, the animal images of the ANID stimuli were selected and cropped in such a way that the animal appeared in the center of the stimulus image.

All of the images were cropped into square shape of 480-pixel side length, thus the display size of each image was 18×18 degree visual field. Half of the images were animal images, and the other half were non-animal (no-target) images. Figure 1 shows examples of the type of animal images (top row) and non-animal images used. The gray-scale images were converted from RGB color using MATLABs (TheMathWorks, Inc., 2010) standard rgb2gray routine, which is a simple linear combination of the RGB color channels (0.2989*R+0.5870*G+0.1140*B).

#### Image statistics

In experiment 1, target and non-target stimuli were composed of two sets of images (animal and non-animal images) from two different image databases (COREL and ANID). The average amplitude spectra of the animal and non-animal images of the two databases were compared. In both of the image databases, the average amplitude spectra were similar between classes. For the COREL database, the mean difference between the average animal and the average non-animal amplitude spectra was 33% of one standard deviation, with a maximum of 65%. For the ANID database, the mean difference between the average animal and the average non-animal amplitude spectra was less than 12% of one standard deviation, with a maximum of 38%.

As the next step, the luminance of all images was calculated. Since the gray-scale images were transformed from the color images by using a simple linear combination of the RGB values (see above), the luminance of the color and gray-scale images were always pairwise the same. The average luminance measures when averaged per image database were extremely similar. The mean difference between the average COREL and ANID luminance measures was 33% of one standard deviation.

Subsequently, the average luminance of the animal and non-animal images of both databases was compared. For the COREL database, the mean difference between the average animal and the average non-animal luminance was 21% of one standard deviation. For the ANID database, the mean difference between the average animal and the average non-animal luminance was merely 2% of one standard deviation.

In general, the difference between animal and non-animal images in the ANID database was smaller than between those in the COREL database. Other than the spectral differences investigated earlier [25], there do not seem to be any major cues to animal presence in both image databases.

### Experimental Procedure

#### Experiment 1

Experiment 1 was a standard 2AFC discrimination paradigm. The participants performed a rapid animal detection task: did the left or the right of the presented images contain an animal? All subjects took part in two sessions. In session 1, their eye movements were recorded; in session 2, their EOG and EEG data were recorded. In one condition the original color images were used, in another condition the gray-scale versions were used.

Target (animal) and distractor (non-animal) images were arranged to the left and right randomly, each located at 10.5 degree horizontal eccentricity, with equal probability in blocks of 100 trials. Each trial contained exactly one target (animal) image. Every subject participated in six blocks, presented in random order exactly once in each session.

Prior to the experiment, subjects were instructed that the animal could appear “either to the left or to the right”. Subjects initiated a trial with a button press. Each trial started with the display of a fixation cross (about 1.2°×1.2°of visual angle) in the center of the screen, in front of a gray background. After a random interval of 800–1600 ms, the fixation cross disappeared, followed by a gap period of 200 ms. After the gap period, the main stimulus image was displayed for 1000 ms, after which it disappeared. Subjects were required to direct their gaze to the side of the target image as quickly and accurately as possible. Before the next trial was initiated, there was a gap period of 400 ms. Subjects were permitted to take a break anytime during the experiment if they so desired. The schematic of the task paradigm is shown in figure 2.

#### Experiment 2

Two kinds of tasks were used in experiment 2, a go/nogo task and a forced choice task. Every subject took part in only one of the tasks, and their EEG data were recorded during the experiment.

Target (animal) or distractor (non-animal) images were shown in the center of the screen randomly, with equal probability in blocks of 120 trials. Half of the stimuli were color images and the other half were gray-scale images, counterbalanced between subjects. Every subject participated in ten blocks in random order exactly once. Prior to the experiment, subjects were instructed that there could be animal or non-animal images appearing on the screen.

In the go/nogo task, subjects initiated a trial by pressing and holding the left button of a mouse. Each trial started with the display of a fixation point (about 1.1°×1.1°of visual angle) in the center of a gray background. After 500 ms, the fixation point disappeared, followed by a random gap interval of 200–400 ms. After the gap period, the stimulus image was displayed for 200 ms. After the stimulus image disappeared, a fixation cross (about 1.1°×1.1°of visual angle) was shown in the center of the screen for a time period of no more than 1000 ms. The display of the cross could be aborted by the subject through releasing the left button of the mouse. Subjects were required to release the button of the mouse when they saw a target as quickly and accurately as possible. Before the next trial was initiated, there was a gap period of 400 ms. In the forced choice task, subjects were required to respond by pressing one of two buttons. One button was assigned to the right hand, the other one to the left hand. Target/non-target button assignments were switched after 5 blocks to balance against possible biases.

Subjects were permitted to take a break anytime during the experiment if they so desired. The schematic of the task paradigm is shown in figure 3.

### Eye movement recording and data analysis

In session 1 of experiment 1, eye movements were recorded using an EyeLink II video-based eye tracking system (SR Research, Canada). The recording frequency was set to 500 Hz. Images were presented on a CRT screen, the active display of which was approximately 40.5 cm wide and 30.5 cm high, at a resolution of 1280×1024 pixels. The refresh rate of the screen was set to 100 Hz.

Stimulus presentation was controlled by a PC running the software written in Matlab, using the Psychtoolbox (Brainard, 1997). The experimental chamber had all major surfaces covered in black and was otherwise dimly lit; the lighting was kept at the same level throughout the experiment. Subjects were seated at 45 cm from the screen with a chin rest to stabilize the head position. At the beginning of each session, subjects were calibrated using the default 9-point calibration algorithm suggested by the manufacturer.

Trials in which the subject had drifted by more than 3° during fixation, or in which the first saccade was smaller than 4.2° were marked invalid. If subjects did not move their gaze onto the target stimulus within 5 s, the trial was marked invalid as well. Finally, saccades faster than 100 ms were considered as errors, possibly driven by the sudden appearance of the stimulus rather than stimulus content, and were also marked invalid. Invalid trials were ignored during further analysis.

### Event-related potential recording and data analysis

In session 2 of experiment 1, the subjects were fitted with an Easy-Cap (Brain Products, Germany). EEG was recorded (using a Brain products system) from 32 channels, based on the international 10–20 system. All electrode sites were referred to an electrode placed on the left mastoid.

In experiment 2, the subjects were fitted with a Quick-Cap (Neuroscan-USA). EEG was recorded (using a Neuroscan system) from 64 channels, based on the international 10–20 system. All electrode sites were referenced to bilateral mastoid electrodes.

Eye movements and blinks were monitored using electrodes placed near the outer canthus of each eye (horizontal electrooculogram HEOG), and above and below the left eye (vertical electrooculogram, VEOG). Inter-electrode impedance levels were kept below 5 kΩ.

EEG was recorded continuously throughout the experiment and was bandpassed from 0.05 to 100 Hz, at a 1000 Hz sampling rate. After completing data collection, the EEG recordings were segmented into 500 ms epochs, starting from 100 ms prior to stimulus onset. Epochs contaminated with artifacts (the threshold for artifact rejection was ±80 μV in all channels) were rejected before averaging. ERPs were filtered digitally prior to peak detection using a bandwidth from 0.1 to 30 Hz.

For session 2 of experiment 1, saccade timing was measured using HEOG with a similar procedure as described by Kirchner and Thorpe [8]: As a first criterion, the difference signal between the left and right EOG electrodes had to exceed an amplitude threshold. Subsequently, the saccade onset time was determined as the nearest signal inflection preceding this point.

Saccadic reaction time was determined as the time difference between the onset of the images and the start of the saccade. Only when the difference signal between the left and right HEOG electrodes exceeded ±30 μV was the trial considered as valid. Then, the saccade onset time was determined as the nearest signal inflection preceding this point. Saccades faster than 100 ms and slower than 250 ms were considered invalid.

Whenever error bars are shown in the figures, these represent one standard error of the mean.

## Results

### Experiment 1

The participants performed a 2AFC animal detection task with the stimuli both in original color and in the gray-scale versions. Images were selected from both the COREL database and the ANID database.

#### Results of saccade measurements

The latency and accuracy of the first saccade were analyzed using an ANOVA design for repeated measures with three factors: image database (COREL vs. ANID), “presence of color” and experiment sessions (session 1 vs. 2). Greenhouse–Geisser adjustments to the degrees of freedom were applied when appropriate.

#### Main effects

Subjects completed the task faster with lower accuracy in session 2 (168 ms, 83%) than in session 1 (176 ms, 87%). The difference between two sessions was significant for saccade latency (*F* (1, 32)  = 15.7, *P*<0.001), and for accuracy (*F* (1, 32)  = 21.8, *P*<0.001). This could result from a trade-off effect between accuracy and latency.

The ANID had higher accuracy than COREL (89% vs. 80%, *F* (1, 32)  = 401.5, *P*<0.001), with nearly identical latencies (172 vs. 173 ms, *F* (1, 32)  = 5.05, *P* = 0.032), see figure 4.

There was no significant overall difference between color and gray-scale images on either saccade latency (170 ms vs. 174 ms) or accuracy (84.5% vs. 85.2%).

#### Interactions: Latency

The difference of latency between the color and gray-scale versions of the ANID database (5.09 ms) was bigger than in the COREL database (3.12 ms). The difference between COREL and ANID databases only reached significance in color images (171.11 ms vs. 168.96 ms, *F* (1, 17)  = 15.78, *P* = 0.001), not in gray-scale images (174.23 ms vs. 174.05 ms, *F* (1, 15)  = 0.04, *P* = 0.85). There was no other significant interaction on latency.

#### Interactions: Accuracy

The accuracy interaction between presence of color and session showed that the color images had higher accuracy (88% vs. 86%, *p* = 0.207, no significant difference) than gray-scale images in session 1, and lower accuracy (81% vs. 84%, *p* = 0.023) in session 2 (*F* (1, 32)  = 8.26, *P* = 0.007). This difference in accuracy was only significant for the ANID database (*F* (1, 32)  = 16.40, *P*<0.001), not for the COREL database (*F* (1, 32)  = 2.44, *P* = 0.128). The interaction of all three factors was also significant (*F* (1, 32)  = 5.56, *P* = 0.025). Statistics are illustrated in figure 4-B.

The accuracy interaction between presence of color and session showed that the accuracy our subjects achieved in session 2 decreased compared to session 1, however this difference was only significant for color images (81% vs. 88%; *F* (1, 17)  = 39.4, *P*<0.001), not for gray images (86% vs. 84%, *F* (1, 15)  = 1.21, *P*>0.05). This difference in accuracy was similar for both image databases, but significant only with the ANID database (*F* (1, 32)  = 16.4, *P*<0.001). The interaction of all three factors was also significant (*F* (1, 32)  = 5.56, *P* = 0.025). Statistics are illustrated in figure 4-B.

#### Results of EEG

The correct-response event-related potential (ERP) of both image databases (COREL vs. ANID) were analyzed separately for the color images and for the gray-scale images. We calculated the frontal brain areas response by averaging the responses for all seven frontal electrodes: FP1, FP2, F3, F4, F7, F8, FZ [1]. Figure 5 showed the pooled ERP waveforms and difference waveforms between color and gray-scale (figure 5A, B), and between COREL and ANID databases (figure 5F) at the frontal brain areas. Here we focus on the analysis of the negative ERP component N1, which was quantified by means of peak amplitude and peak latency, with peak amplitude measured relative to baseline and peak latency measured in relation to stimulus onset.

The amplitudes and latencies of N1 were analyzed using an ANOVA design for repeated measures with image database (COREL vs. ANID) and electrodes as within-subjects factors, and the “presence of color” as between-subjects factor. Greenhouse–Geisser adjustments to the degrees of freedom were applied when appropriate.

The color images induced greater N1 amplitude (−5.62 μV) than the gray-scale images (−4.27μV), *F* (1, 32)  = 7.32, *P* = 0.007, with shorter latency (196.43 ms vs. 209.05 ms), *F* (1, 32)  = 5.69, *P* = 0.018, figure 5 E. We plotted topographic maps for the difference waves between color and gray-scale images separately per database. The ERPs were integrated across 20 ms time windows, from 120 ms to 259 ms. N1 was located mainly in the frontal brain areas, and this difference began at around 170–180 ms. Comparing figures 5 C and D, the difference between color and gray-scale images can be seen to emerge earlier with the ANID database than with the COREL database.

The N1 amplitude of COREL (−4.97 μV) and ANID (−4.92μV) were very similar with very close latency (203.17 ms vs. 202.30 ms). There was no interaction for the N1 amplitude and latency.

In Experiment 1, we investigated the effects of color and database on human rapid animal detection performance by means of a 2AFC paradigm. We chose eye movements as our response modality, as they are fast and usually very precise. However, some of the effects we found may be specific to eye movements, as they are easily influenced by the appearance of the task. Additionally, the visual processing in the direct comparison of two images may differ from conditions where only one image is shown at a time. To investigate this, we devised a second experiment, which included a different response modality as well as a different experimental paradigm.

### Experiment 2

The participants performed a go/nogo or a forced choice animal detection task with the stimuli both in original color and in the gray-scale versions. Images were selected from both the COREL database and the ANID database. In this experiment, subjects used button press instead of eye movements.

#### Behavioral results

The accuracy and response time (Rt) of the subjects were analyzed using an ANOVA design for repeated measures with two factors: image database (COREL vs. ANID) and “presence of color”. Greenhouse–Geisser adjustments to the degrees of freedom were applied when appropriate.

Color images had higher accuracy and slower response time than gray-scale images both in go/nogo task (accuracy: *F* (1, 15)  = 552.6, *P*<0.001; response time: *F* (1, 15)  = 14.5, *P* = 0.002) and in forced choice task (accuracy: *F* (1, 15)  = 599.5, *P*<0.001; response time: *F* (1, 15)  = 6.18, *P* = 0.024).

The COREL database had higher accuracy than the ANID database in both tasks: go/nogo: *F* (1, 15)  = 29.4, *P*<0.001; forced choice: *F* (1, 15)  = 19.3, *P*<0.001), with almost the same response time. See figure 6. Table 1 lists the details of the accuracy and response time in both go/nogo and forced choice tasks.

**Table 1:** Performance of go/nogo and forced choice task in experiment 2.

	Accuracy (%)				Response time (ms)			
Database	COREL		ANID		COREL		ANID	
Hue	Color	Gray	Color	Gray	Color	Gray	Color	Gray
**Go/nogo**								
Animal	94.6	92.6	89.8	85.4	367.7	339. 9	366.3	340.9
Non-animal	93.6	90.0	92.3	93.9	-	-	-	-
**Forced choice**								
Animal	93.9	93.0	89.6	88.2	359.5	342.4	357.9	335.4
Non-animal	92.2	90.8	90.7	90.9	367.0	337.8	361.3	335.2

#### Comparing between go/nogo and forced choice task

The accuracy in both tasks was very high, both more than 90%. In the forced choice task, animal and non-animal image trials had very similar accuracy (91.2% vs. 91.15%). But in the go/nogo task, the accuracy of non-animal trials was higher than animal trials (92.45% vs. 90.6%). The response time of the detection of animal stimuli in the forced choice task was faster than in the go/nogo task (see table 2).

**Table 2:** Mean accuracy and response time of go/nogo and forced-choice task in experiment 2.

Task	Animal		Non-animal	
	Accuracy (%)	Rt (ms)	Accuracy (%)	Rt (ms)
Go/nogo	90.6	358.3	92.45	null
Forced choice	91.2	348.4	91.15	350.3

#### Results of EEG

The correct-response ERPs of target (animal) and distractor (non-animal) images were computed separately. These two ERPs were compared by subtracting the target ERPs from the distractor ERPs. For both go/nogo and 2AFC tasks, the ERPs of the two image databases (COREL vs. ANID) in the two hue conditions (color vs. gray-scale) were analyzed separately.

The ERPs of animal and non-animal stimuli diverged at around 150 ms after stimulus onset, an effect which was particularly clear at frontal electrodes, thus we calculated the frontal brain areas response by averaging the responses for all seven frontal electrodes: FP1, FP2, F3, F4, F7, F8, FZ (Thorpe, Fize, & Marlot, 1996).

The peak amplitude and peak latency of P1 and N1 were analyzed using an ANOVA design for repeated measures with “animal/non-animal”, “presence of color”, image databases (COREL, ANID) and electrodes as within-subjects factors. Greenhouse–Geisser adjustments to the degrees of freedom were applied when appropriate.

The N1 amplitude with non-animal stimuli was bigger than with animal stimuli both in the Go/nogo task (−11.12 μV vs. −9.22 μV, *F* (1, 15)  = 14.06, *P* = 0.002) and in the Forced choice task (−8.26 μV vs. −7.25 μV, *F* (1, 17)  = 12.03, *P* = 0.003). The two ERPs diverged to a statistically significant amount from 182 ms (*p*<0.05) for the Go/nogo task and 179 ms ((*p*<0.05) for the Forced choice task. This results are similar to those reported by Thorpe, see figure 7, 8 (A) (Thorpe, Fize, & Marlot, 1996).

However, the difference between animal and non-animal stimuli with the COREL database was bigger than with the ANID database (Go/nogo: *F* (1, 15)  = 10.13, *P* = 0.002; Forced choice: *F* (1, 15)  = 6.20, *P* = 0.001), figure 7, 8(B, C). Especially, this difference was statistically significant from 179 ms (go/nogo), 147 ms (forced choice) for the COREL-color images, 171 ms (go/nogo), 183 ms (forced choice) for the COREL-gray-scale images (*p*<0.05) on go/nogo and 2AFC tasks separately, however no similar effects were found on the images of the ANID database (paired t-test at each time point of the difference ERPs between −100 ms to 400 ms; figure 7D, 8D).

For both animal and non-animal stimuli, the color images induced larger and earlier amplitudes than gray-scale images, however the difference was statistically significant only on the go/nogo task: P1: *F* (1, 15)  = 5.36, *P* = 0.035; N1: *F* (1, 15)  = 5.75, *P* = 0.018, figure 9. On the forced choice task, color images induced larger N1 than gray-scale images on animal trials with the ANID database (−8.16 μV vs. −7.79 μV, *P* = 0.001).

The COREL database induced larger P1 and smaller N1 than the ANID database: go/nogo task: P1: *F* (1, 15)  = 8.08, *P* = 0.012; N1: *F* (1, 15)  = 3.9, *P*<0.001); forced choice task: P1: *F* (1, 15)  = 4.67, *P* = 0.032; N1: *F* (1, 15)  = 11.5, *P* = 0.01). Table 3 listed the detailed data both go/nogo and forced choice tasks.

**Table 3:** P1 and N1 amplitude and latency of go/nogo and forced choice task in experiment 2.

Task	Go/nogo				Forced choice			
	amplitude (uv)				amplitude (uv)			
	latency (ms)				latency (ms)			
Database	COREL		ANID		COREL		ANID	
Hue	Color	Gray	Color	Gray	Color	Gray	Color	Gray
**P1**								
Animal	2.83	2.44	2.33	2.25	3.73	3.61	3.21	3.19
	127.8	133.3	120.4	125.8	132.1	128.4	123.9	125.4
Non-animal	2.48	1.57	2.14	1.86	3.25	3.02	3.01	2.59
	117.7	118.3	119.9	116.8	120.1	124.2	119.1	119.0
**N1**								
Animal	−8.82	−8.31	−10.3	−9.46	−6.44	−6.59	−8.24	−7.74
	217.6	224.6	221.0	222.6	212.2	214.1	221.5	225.3
Non-animal	−11.02	−10.94	−11.37	−11.21	−7.95	−7.60	−8.62	−8.86
	222.8	222.8	218.5	228.8	218.0	227.3	211.3	216.9

## Discussion

We discovered several significant differences in behavioral performance between color and gray-scale images. However, all these differences were rather small and it is difficult to exclude possible trade-offs between speed and accuracy as their cause. In contrast, we found that in all tasks color images induced greater N1 at earlier time points than the gray-scale images. The ERP results of go/nogo and forced choice tasks were similar to those reported by Thorpe and colleagues [1]: the N1 amplitude with non-animal stimuli was bigger than with animal stimuli. However, this difference was bigger for the COREL images than within the ANID images.

In addition to these general findings, we observed several differences between the different paradigms. Color did not increase the performance in the 2AFC task, but increased both accuracy and response time in the go/nogo and forced choice tasks. The ANID images had higher accuracy than the COREL images in the 2AFC task, but had opposite effects in go/nogo and forced choice tasks. The ANID and COREL images showed significant differences on ERPs in the go/nogo and forced choice tasks, but not in the 2AFC task. With the ANID images, the difference between color and gray-scale version images was more pronounced than with the COREL images. This bigger difference within the ANID database may result from the way the ANID database was constructed: animal and no-animal images were taken from the same original scene, resulting in very similar color distributions. In the extreme case, the only noteworthy color distribution difference between two images would be the animal itself (e.g. green grass and blue sky vs. green grass, blue sky and a brown or black-and-white cow). This might make color a more diagnostic feature for the ANID database, as the pairwise unrelated images from the COREL database naturally exhibit more variable color distributions. However, the images of the ANID database were taken in the natural habitats of the animals, and therefore exhibit a higher ecological validity than the COREL images. The nature of the difference varies with the different tasks: (a) In the 2AFC task, the saccade latency of the color images was smaller than that of the gray-scale images and this difference was larger with the ANID database than with the COREL database. (b) In the 2AFC task, the ERP difference waves between color and gray-scale images emerged earlier with the ANID database than with the COREL database. (c) In the forced choice task, during animal trials, the difference of the N1 amplitudes between color and gray-scale images was bigger with the ANID database than the COREL database.

### Effects of color

Color appears as a more relevant feature in the processing required to detect whether there is an animal in one given image (animal or non-animal, experiment 2) than the processing required to identify where the animal is in two images (animal and non-animal, experiment 1). Table 4 showed the detailed comparison between color and gray images in different tasks.

**Table 4:** The effects of color on different tasks.

	Accuracy	Response time	P1		N1	
			amplitude	latency	amplitude	latency
2AFC	ns	ns	-	-	color>gray*	color<gray*
Go/nogo	color>gray*	color>gray*	color>gray*	color<gray	color>gray*	color<gray*
Forced choice	color>gray	color>gray*	color>gray*	color<gray*	color>gray	color<gray*

* the mean difference is significant at the.05 level.

Color might be more or less “diagnostic” in the recognition of certain object categories [39], [41], [47]. In other words, the use of color features to determine whether an item belongs to a category might depend on the pertinence of color in identifying objects from that category [37]. For animal categorization, the color showed different effects on different tasks, perhaps because the effect of color was masked by other features in the recognition of animal, for example global scene cues and context congruence [24]. In experiment 1, both animal and non-animal images were shown simultaneously on the screen, which supplied more contextual information for the detection than when only one image was shown on the screen in experiment 2.

The effect of color on the recognition of animals was confirmed by the EEG data. Colorful images induced larger activation at an earlier time point in the EEG in both experiments. Thus, we conclude that color images induce more neural activity than gray-scale images, at least during tasks requiring the recognition of animals. The effects were stronger in the ANID database.

It has been suggested that early differences in EEG signals (prior to 150 ms) may reflect systematic differences in low-level stimulus properties common to objects in a given category – such as spatial frequency content, simple spatial patterns and textures [16], [23]. The early ERP components, P1 and N1, have been associated with initial sensory input, bottom-up processing [48], and selectively attending to non-spatial stimulus features, such as color [49], [50].

On the other hand, the ERP components elicited by stimuli having the attended feature were interpreted as reflecting the attentional facilitation of processing in feature-specific “channels” of visual input [51], [52]. The amplitude of P1 has been shown to represent the cost of shifting the attention to the place where the target stimulus is located [53]. The amplitude increase in the N1 has been taken as evidence that attention allows for more extensive analysis of visual information, such as color [54]. Thus, we suggest that color images might attract more attention, which modulates or facilitates the processing of animal detection compared to gray-scale images.

### Effects of image database

Image database appears as a relevant feature in our experiments. The ANID images were classified with higher accuracy than those from the COREL database in the 2AFC tasks but lower accuracy in the go/nogo and forced choice tasks. It has been shown that contextual cues [24] and context congruence [25] are important for animal categorization. As we discussed previously, in experiment 1, animal and non-animal images were displayed simultaneously on the screen, which supplied more contextual information for the recognition of the animal. In contrast, in experiment 2, animal and non-animal images were displayed separately in different trials. The non-animal images of the ANID database are crops taken from the spatial neighborhood of the animal crops, which probably reduced the difference between animal and non-animal images and may have resulted in increased difficulty of recognition. The EEG results provided further confirmation. In experiment 2, we replicated the results reported in Thorpe's paper [1] that the N1 amplitude with non-animal stimuli was bigger than with animal stimuli. However, the difference within the COREL images was bigger than within the ANID images. It has been suggested that the enhanced negativity observed on no-go trials, “could reflect a role for frontal areas in inhibiting inappropriate behavioral responses” [1]. Thus, we conclude that the non-animal images of the COREL database on average induced larger activation to inhibit “inappropriate behavioral responses” than the non-animal images of the ANID database, which resulted in better performance of the COREL database in go/nogo and forced choice tasks.

It has been shown that the differential activity between targets and non-targets does not simply reflect systematic “low-level” visual differences or the extraction of basic visual properties, but correlates with the status (target or distractor) of the stimulus [16]. However, in our experiments, the “low-level” visual differences between image databases most likely influenced the differential activity between targets and non-targets. Table 5 showed the detailed comparison between the ANID database and the COREL database on different tasks.

**Table 5:** The effects of database on different tasks.

	Accuracy	Response time	P1		N1	
			amplitude	latency	amplitude	latency
2AFC	COREL<ANID*	COREL>ANID*	-	-	ns	ns
Go/nogo	COREL>ANID*	COREL>ANID	COREL>ANID*	COREL>ANID*	COREL<ANID*	COREL<ANID*
Forced choice	COREL>ANID*	COREL>ANID	COREL>ANID*	COREL>ANID*	COREL<ANID*	COREL>ANID

* the mean difference is significant at the.05 level.

In our research, the role of low level image features for the recognition of objects in natural scenes was investigated. We found that rapid animal detection is not unaffected by color, but that the influence of color may be masked by other factors, such as contextual cues and context congruence. However, even when the ‘other factors’ masked the effects of color in the measured subjects during the animal detection task, color images still induced more neural activity than gray-scale images. The controversial conclusions in previous literature about the effects of low level features on object recognition might be due to different image databases, tasks and measurement modalities used in the experiments. Therefore, we conclude that the ANID image database is better suited for psychophysical research than other databases commonly used, especially those based on the popular Corel image collection. With the ANID database, low-level features are better controlled, and fewer global differences exist between target and non-target images.
